# Structural characterization of the saxitoxin-targeting APTSTX1 aptamer using optical tweezers and molecular dynamics simulations

**DOI:** 10.1371/journal.pone.0222468

**Published:** 2019-11-07

**Authors:** Nathalie Casanova-Morales, Nataniel L. Figueroa, Karol Alfaro, Felipe Montenegro, Nelson P. Barrera, J. R. Maze, Christian A. M. Wilson, Pablo Conejeros

**Affiliations:** 1 Instituto de Física, Pontificia Universidad Católica de Chile, Santiago, Chile; 2 CIGREN. Instituto de Biología. Facultad de Ciencias. Universidad de Valparaíso, Valparaíso, Chile; 3 Department of Physiology, Faculty of Biological Sciences, Pontificia Universidad Católica de Chile, Santiago, Chile; 4 Departamento de Bioquímica y Biología Molecular, Facultad de Ciencias Químicas y Farmacéuticas, Universidad de Chile, Santiago, Chile; 5 Facultad de Artes Liberales, Universidad Adolfo Ibáñez, Santiago, Chile; LAAS-CNRS, FRANCE

## Abstract

Optical tweezers have enabled the exploration of picoNewton forces and dynamics in single–molecule systems such as DNA and molecular motors. In this work, we used optical tweezers to study the folding/unfolding dynamics of the APTSTX1–aptamer, a single-stranded DNA molecule with high affinity for saxitoxin (STX), a lethal neurotoxin. By measuring the transition force during (un)folding processes, we were able to characterize and distinguish the conformational changes of this aptamer in the presence of magnesium ions and toxin. This work was supported by molecular dynamics (MD) simulations to propose an unfolding mechanism of the aptamer–Mg^+2^ complex. Our results are a step towards the development of new aptamer-based STX sensors that are potentially cheaper and more sensitive than current alternatives.

## Introduction

Harmful algal blooms contain toxins that, when bio-accumulated by bivalve molluscs, can be fatal upon human consumption. Among these toxins, saxitoxin (STX) is particularly dangerous due to its binding-affinity to sodium channels in voltage-excitable cells [1]. Through this, STX causes asphyxia from diaphragm paralysis in less than an hour, with a lethal oral dose in humans as low as 1–4 mg [2].

To avoid poisoning, STX concentrations are monitored regularly by injecting mice with a shellfish extract and then measuring their time of death [3, 4]. Although other methods of STX detection are available, they require expensive equipment and lack the robustness and sensitivity of the mouse bioassay [3, 5–7].

Recently, a new method was used to quantify nanomolar concentrations of STX through conformational changes of an aptamer [8–10]. These are single stranded oligonucleotides (ssDNA or RNA) that bind to a target molecule with high affinity and specificity [11]. Aptamers can be created for a specific ligand through a process called “Systematic Evolution of Ligands by Exponential Enrichment” (known as SELEX) [12, 13], in which the initially randomized aptamers that bind to the target are isolated and amplified using polymerase chain reaction [14] several times. In principle, this technique allows the production of aptamers that bind to any specific ligand [11, 15]. Although aptamer-target binding affinity is comparable to that of monoclonal antibodies [16], aptamers are cheaper to produce and are more stable, have a longer shelf life, and are capable of returning to their active state after being denatured by pH or temperature [17]. All these characteristics make aptamers a promising tool for a variety of applications, which has motivated their study [18, 19].

The development of a aptamer–based biosensing platform for SXT would benefit from understanding conformational changes in aptamers, as more sensitive aptamers could be designed. In this work, we studied the affinity and mechanical stability of the APTSTX1 aptamer binding to SXT and magnesium ions (Mg^+2^, a known DNA stabilizer [20]) at the single-molecule level using optical tweezers. This technique (which recently earned their inventor, Arthur Ashkin [21], the Nobel prize in 2018) uses laser light to trap and manipulate particles, which can be used to study the mechanical properties of biomolecules. Using optical trapping, we were able to pull on single aptamers and observe their (un)folding in different media (with and without Mg^+2^ or STX) and show the stabilization of the aptamer upon binding. This work was complemented with molecular dynamics (MD) simulations to propose an unfolding mechanism of the aptamer–Mg^+2^ complex, which is in agreement with the single-molecule experiments.

## Materials and methods

**Fig 1:**
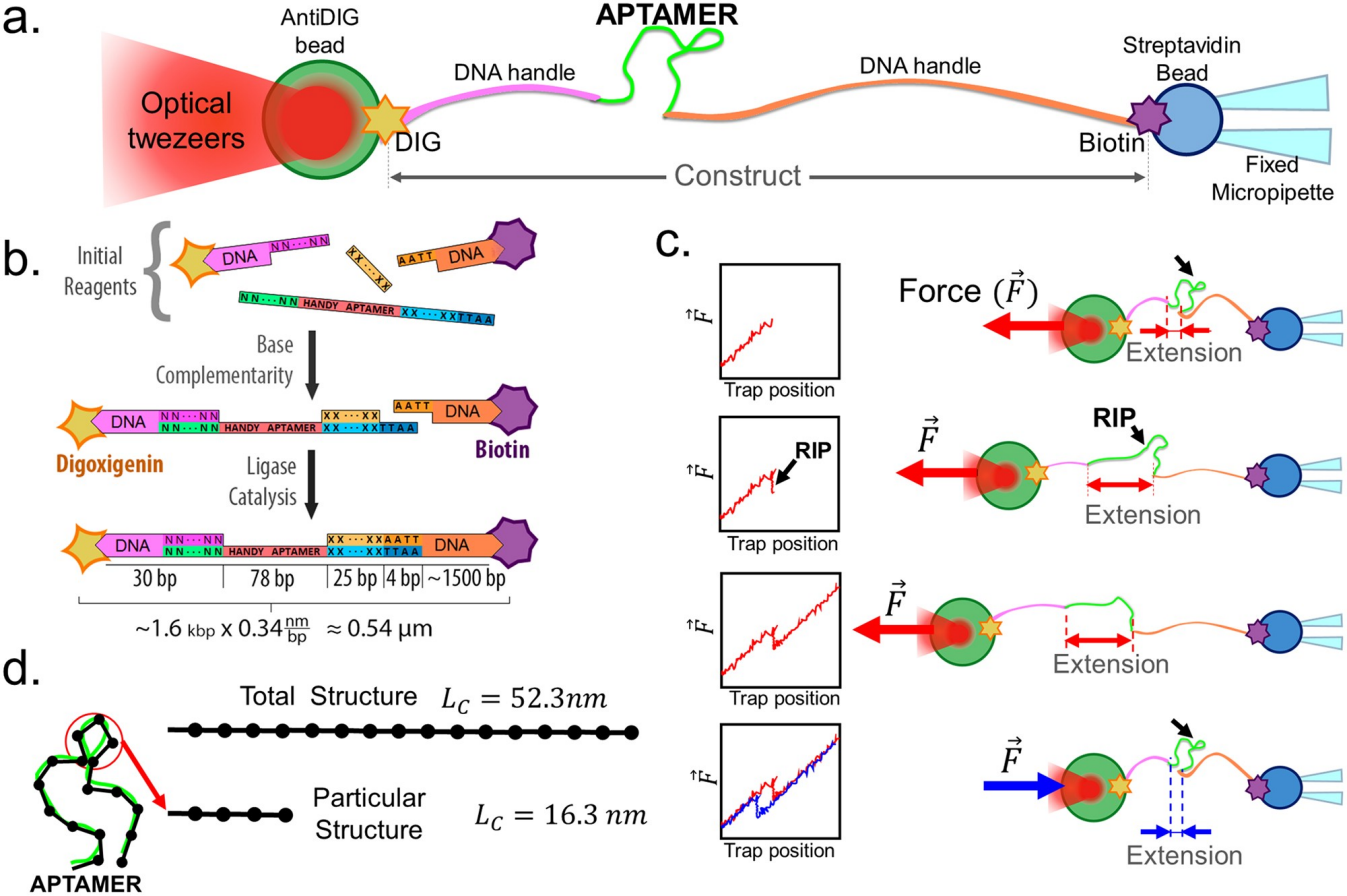
Experimental setup. (a) Step by step diagram for the synthesis of the construct. The different colors designate different nucleotide sequences. The “N” and “X” symbols represent complementary bases and “A” and “T” adenine and thymine, respectively. The star–like shapes represent digoxigenin and biotin molecules. (b) Optical tweezers experimental set–up. The final construct of APTSTX1 aptamer is attached to the beads by means of DNA handles. These DNA handles are connected to both ends with digoxigenin (DIG) and biotin (antidigoxigenin–coated beads and streptavidin–coated beads, respectively). The streptavidin bead is held in place by suction through a micropipette and it has a diameter of 2 *μ*m, and the 3 *μ*m DIG bead with the final construct (DIG–DNA handle–Aptamer–DNA Handle-Biotin) is captured by the optical trap. This system is inside a laminar flow chamber. (c) Force extension hysteresis curves obtained by stretching (red) and relaxing (blue)). Data shown were collected at a 50 Hz sampling rate and a pulling speed of 100 nm s^−1^. These data are taken under control (CNT) conditions. (d)This figure illustrates the measurements of contour length. These correspond to smaller structures (for example 16.3 nm) that are folded once or more in the aptamer. Total length of aptamer is 52.3 nm.

In order to pull on single aptamers, we chemically attached DNA-handles to the ends of them. These handles connect the aptamer to micron-sized polysterene microsphere (as shown in Fig 1a). In a pulling experiment, one of the microspheres was fixed to a micropipette using suction, while the other microsphere was controlled using an optical trap in order to pull the aptamer. The tension on the ends of the aptamer was measured optically as the aptamer was pulled.

### Construction of DNA handles to study the single–molecule aptamer

Two DNA handles were attached at the ends of the aptamer in order to pull it (see Fig 1b). These two handles were 30 bp (∼ 10*μ*m) and 1500 bp (∼ 500*μ*m) long, and ensure that the aptamer (∼ 0.54*μ*m long) was located between the two microspheres.

These handles were attached to the aptamer by using two ssDNA adapter segments that were added to the ends of the APTSTX1 aptamer (5’– AATT GC ATC TGT GCG GTA TTT CAC ACC GT GGT ATT GAG GGT CGC ATC CCG TGG AAA CAT GTT CAT TGG GCG CAC TCC GCT TTC TGT AGA TGG CTC TAA CTC TCC TCT GCC AGC AAG ACG TAG CCC AGC GCG TCG GCC—3’). The 5’ handle was obtained by PCR amplification of a 3427 bp DNA fragment from the Lambda Phage using biotinylated primers Forward (5’– / 5Biosg / ACC TGC CAG AAC ATT CAG CTG 3’) and Reverse (5’/ 5Biosg / AAC GAC TAT GCC CTT ACA GCA G 3’). The amplicon was then cut into two fragments of similar size using EcoRI enzymatic digestion (Promega, US) for 3 hours at 37 ºC. Both 5’ biotinylated fragments were purified from a 1% Agarose gel (UltraClean DNA Purification Kit, Qiagen, Germany) and ligated with T4 ligase (Promega, US) to the 5’ adapter of the aptamer by adding a complementary oligo (5’–ACGGTGTGAAATACCGCACAGATGCGCATG–3’) (Fig 1a). The 3’ handle was obtained by adding a 5’ Digoxigenine (DIG) labelled oligo (5’/ DIGN / GGC CGC CGC GCT CTA GGG GCT CGT CTT GGC–3’) that is complementary to the 3’ adapter in the aptamer. After ligation and binding, the construct (DIG–DNA Handle–Aptamer–DNA Handle–Biotin) was purified from a 1% agarose gel electrophoresis by cutting the expected size of the construct and purifying it with a gel extraction kit (Qiagen, USA). All oligos were purchased from the IDT DNA company, US.

### Single–molecule assays

A miniTweezers instrument [22] was used for the single–molecule experiments. The miniTweezers setup includes a microfluidic cell with three different channels, a micropipette in the central channel and a laser trap for handling the microspheres (Spherotech, US) (Fig 1a). Before an experiment, Anti–Digoxigenin antibody (Anti–DIG) coated microspheres were incubated at room temperature for 30 min in a solution of: 9.5 *μ*L reaction media with 0.5*μ*L DNA (0.1 − 0.5*μ*M) and 5*μ*L of Anti–DIG polystyrene microspheres. The streptavidin coated microspheres were diluted in 1 mL of each reaction media for every 2 *μ*L of microspheres.

The streptavidin and construct-incubated coated microsphere solutions are placed in different microfluidic channels, to avoid binding. Both of these channels are connected to a central channel (where the micropipette is) by glass tubes, allowing a small number of microspheres to flow into it. Note that different sized microspheres were used for the different coatings, in order to distinguish them easily.

The pulling experiment begins by using the optical trap to place a streptavidin-coated microsphere on the micropipette in the central microfluidic channel, where it is held in placed by suction. Then, an AntiDIG–coated microsphere that had been incubated with the final construct (DIG–DNA handle–Aptamer–DNA Handle-Biotin), was optically trapped and moved near the micropipette–trapped microsphere.

In proximity, both microspheres bind to the DNA handles located at the ends of the construct (Fig 1b). Then the optically–trapped microsphere is moved away from the micropipette–trapped microsphere, pulling the construct. While pulling, the force between the spheres is measured through deflections of the trapping laser.

Under force the molecule can unfold, rapidly changing its extension (Fig 1c), we obtained and analyzed the force where these events happen (See S1 Fig). The spheres were separated in a ramp-like fashion until a fixed distance that was chosen to be far compared to distances where rips were observed. Then, the optically trapped bead was brought back to the starting position with the same speed mentioned previously and the pulling was repeated. The molecules that we studied were pulled at a velocity of 100 nm s^−1^ with a trap stiffness of 0.1 pN nm^−1^, giving a constant loading rate of 10 pN s^−1^. Finally, we calculated the length of the (un)folded structures that were observed by modelling the aptamer like a worm-like chain (WLC).

Three different assays were done to study the (un)folding properties of the APTSTX1 aptamer: phosphate buffer solution (PBS)–only (control condition; CNT), PBS with 3 mM MgCl_2_ and PBS with 38 *μ*M STX. In order to assure that a single molecule was being observed, we pulled to high forces (∼ 70 pN) in order to see overstretching [23]. If two or more molecules were present, overstretching occurred at a much higher force because the load is distributed among the different molecules, which was addressed by releasing the microspheres and trapping a new pair.

### Worm like chain model and analysis

The WLC model is commonly used to describe the behaviour of flexible polymers in a thermal bath. A stretched polymer tends to pull on its ends, as thermal forces try to randomize the alignment along its chain segments. The magnitude of this force is given by [24]:

$$\begin{array}{c} F=\frac{k_{B}T}{L_{P}}[\frac{1}{4}(1-\frac{x}{L_{C}}{)}^{-2}-\frac{1}{4}+\frac{x}{L_{C}}]\; \end{array}$$ (1)

Where *L*_*P*_ is the persistence length, a value related with the material of the chain, x is the end–to–end extension and *L*_*C*_ is the contour length.

In our case, the aptamer can be thought of a chain as illustrated in Fig 1d. The contour length *L*_*C*_ of the whole aptamer has an expected value of 52.3 nm (78 bases × 0.676 nm base^−1^ [25]). From this it is clear that 3-D structures formed by the aptamer will have a contour length smaller than 52.3 nm (as illustrated in Fig 1d).

In order to obtain *L*_*P*_ for the aptamer, WLC curves were fitted to the rip force-extension data, but since all the rips occurred at relatively small forces, *L*_*P*_ could not be precisely determined (note in Eq 1 the WLC force is rather insensitive to *L*_*P*_ for small *F* because, *δF*/*δL*_*P*_ ∝ *F*). For this reason, we used a previously reported value for ssDNA of *L*_*P*_ = 0.75 nm [23] for our calculations. With this, it was possible to obtain the contour length of the structures that were unfolded during the pulling.

Our choice of using the WLC model to describe ssDNA is motivated by the previous reports that validate it [20] and its extensive use in the field [26], that allows us to present results that are directly comparable with previous findings.

We can also derive good extensions directly from rip raw data without knowing trap stiffness because the discontinuous curves (before and after the rip) pass through the same force, making the handle extensions and bead compliances the same (before and after) so we only need to look at the difference in trap positions to get the extension change due to the aptamer (see S1 Fig).

### Comparison criteria between histograms

To quantify the difference between the data that was taken in different media, we used a Mann-Whitney-Wilcoxon rank sums nonparametric test. This test was chosen because the distributions obtained are not well-modeled by Gaussian distributions (all distributions were rejected by a one-sample Kolmogorov-Smirnov normality test at a 0.05 significance level). Additionally, the Mann-Whitney-Wilcoxon test has been used previously in similar work [27]. P-values presented in this report correspond to that of the context-relevant single-sided test. We considered *p* < 0.05 to be statistically significant [28].

### Three–dimensional model, molecular dynamics (MD) and steered molecular dynamics simulations (SMD) of the APTSTX1 aptamer

In order to compare and understand the mechanical behaviour of the aptamer, we produced a 3D model of the naturally folded state of the APTSTX1 aptamer by running MD simulations using the NAMD simulation package [29] with the CHARMM36 force field using optimum RNA parameters [30]. The initial 3D model of the APTSTX1 aptamer was created using 3DNA software [31] and was based on a 2D MFOLD-simulation [32]. The aptamer model was then placed in a grid size of 158 × 161 × 398 Å^3^ with 451116 TIP3 water molecules. The cut–off distance for non–bonded van der Waals interactions was set to 12 Å with a switching function of 10 Å. Electrostatic interactions were calculated using the particle mesh Ewald method [33]. Later, the 50 ns MD simulations of the APTSTX1 aptamer were performed with different Mg^+2^ atoms in the grid (0, 15, 150 and 1500 Mg^+2^ atoms) in the NPT regime (constant temperature, pressure, and number of particles) at 300°K and with a Shake algorithm for geometrical constraints [34]. The temperature was controlled by Langevin dynamics with a damping coefficient of 5 ps^−1^ a pressure equivalent to that of a Langevin piston [35], and an integration time step of 2 fs. A series of measurements were implemented in VMD [36] to quantify the folding of the aptamer, these included the radius of gyration (Rg), the root–mean–squared deviation (RMSD) of all heavy atoms, and the amount of Mg^+2^ ions near the aptamer.

Secondly, using the final folded APTSTX1 aptamer files after 50 ns MD simulations, we applied pulling forces via SMD simulations to look for the unfolding of structures with the CHARMM36 force field without periodic boundary conditions. Pulling at constant velocity was done following the potential energy measurement given by:

$$\begin{array}{c} U=0.5k{\left[vt-\left(\vec{p}-{\vec{p}}_{0}\right)d\right]}^{2} \end{array}$$ (2)

where *v* represents the constant velocity of 0.005 Å ps^−1^, within the range of standard pulling conditions used in SMD for macromolecules [37], *k* represents the spring constant of 69.48 pN Å^−1^ that was used between the dummy and the pulled SMD atom (C2 of 5’ timine), *t* represents the time for each SMD simulation (10 ns), $\vec{p}$ and ${\vec{p}}_{0}$ represent the current and initial position of the SMD atom, and *d* represents the direction of pulling which was calculated for every simulation based on the position of the carbons located at both end of the DNA strand (C2 of the 3’ guanine and SMD atom). In order to study the stabilization effect of Mg^+2^, we simulated five independent pulling experiments with 0, 15, 150 and 1500 Mg^+2^ atoms in the cell. The force-extension curves of the stretched aptamer were calculated, where the length was defined as the distance between the carbons located at both end (described above) of the DNA strand.

## Results and discussion

**Fig 2:**
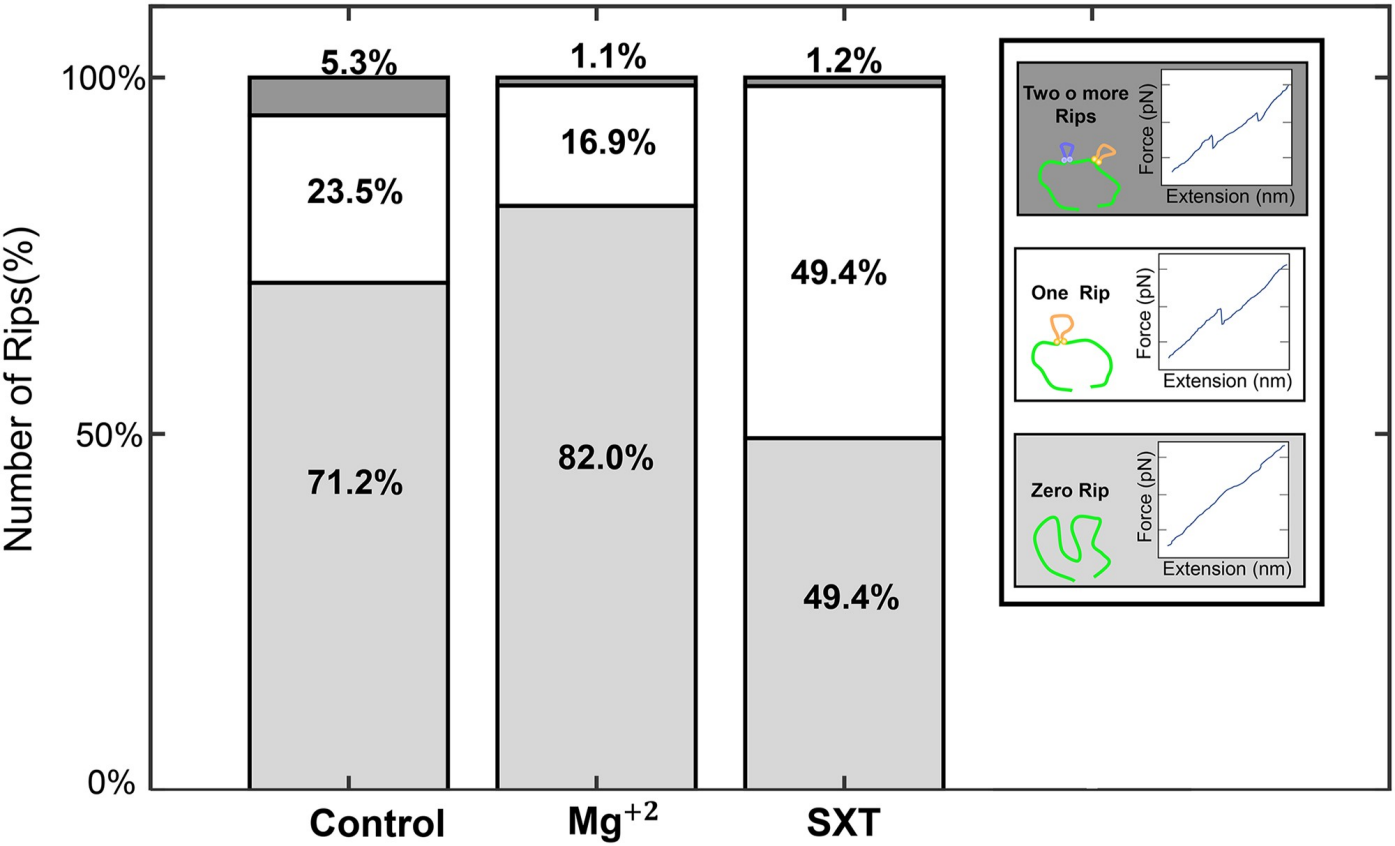
Abundance of rips in the APTSTX1 aptamer with Mg^+2^ and SXT. Number of rips observed in unfolding state under the different experimental conditions. Light grey represents the percentage for zero number of rips, white represent 1 rip in the force vs extension graph, ie, only one structure is unfolded as shown in the illustration and dark grey represents more than 1 rip, ie, two or more structure are unfolded as shown in the illustration. Total number of experiments considered were 132 for CNT, 77 for SXT, and, 178 for Mg^+2^.

In order to characterize the structure of the STX aptamer with optical tweezers, we observed that after 132 pulling experiments of a single APTSTX1 aptamer under PBS–buffer–only, control conditions (CNT), 71.2% of the cases did not present folding or unfolding (Fig 2 Control; a representative curve is shown in S2 Fig). This rate of non-observation is quite high compared to that of bigger molecules usually studied with optical tweezers, suggesting a small binding energy, consistent with relatively short hairpins of the aptamer.

**Fig 3:**
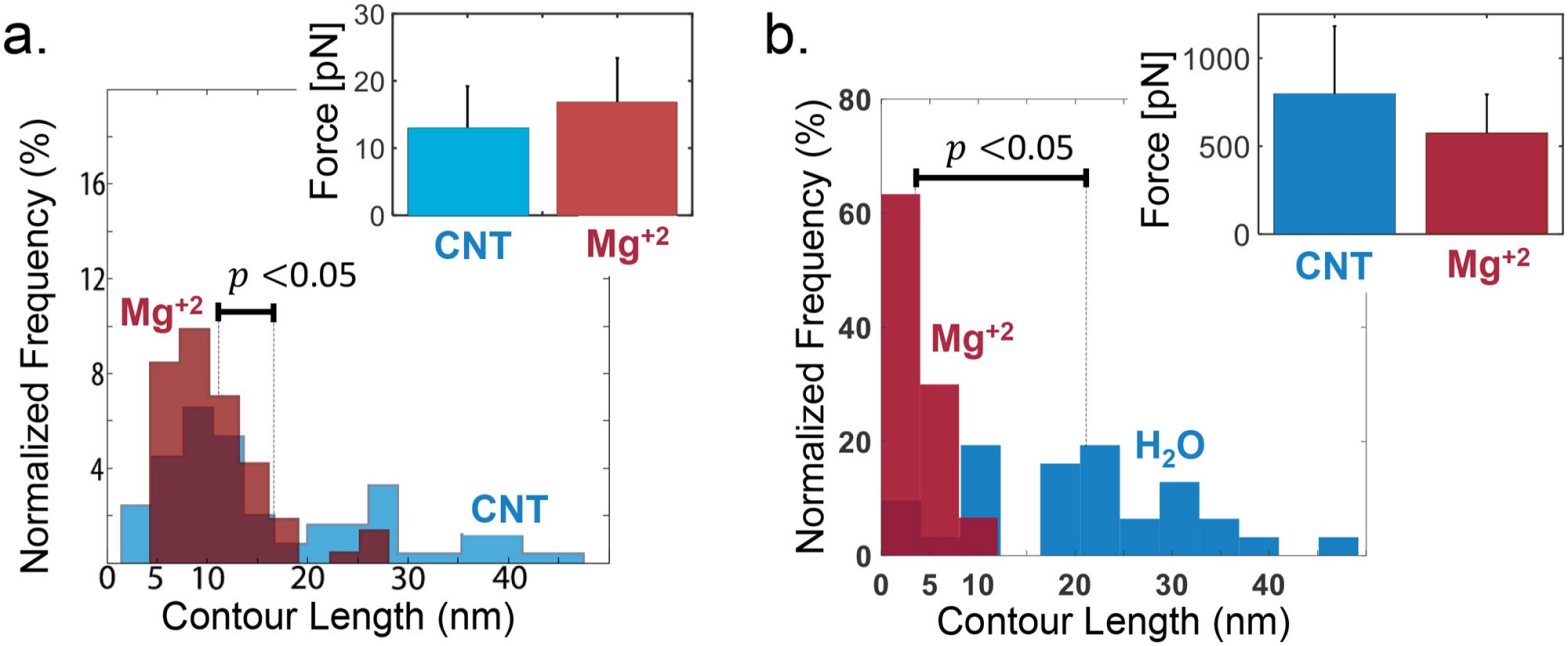
APTSTX1 aptamer stabilization with Mg^+2^. (a) Comparison of the contour length histograms for the control (CNT, blue) and Mg^+2^ environment (red). Total number of rips (Unfolding and Refolding) considered are CNT: 79, Mg^+2^: 71. The mean of distribution for Mg^+2^ treatment (10.8 nm) is clustered on a particular size and showing a significant difference (*p* < 0.05). Inset, rupture force results in CNT (blue) and Mg^+2^ environment (red). Total number of rips considered (only Unfolding) are 45 and 43 for the control and Mg^+2^ environments, respectively. (b) Comparison of the contour length histograms for the control (CNT, blue) and Mg^+2^ environment (red). Total number of rips considered (only Unfolding) CNT: 31, Mg^+2^: 35, showing a significant difference (*p* < 0.05). Inset, rupture force for the APTSTX1 aptamer via SMD simulations in CNT (blue) and Mg^+2^ environment (red).

To observe the aptamer in a more stable structure we used Mg^+2^. This DNA and RNA stabilizer [38], was added into the solution in an attempt to stabilize the aptamer structures that were being unfolded. In spite of this, out of the 178 experiments, 82% of the cases did not present folding and unfolding (Fig 2 Mg^+2^). Furthermore, the contour length distribution of the APTSTX1 aptamer under Mg^+2^ (average 10.8 nm) showed significantly smaller lengths (*p* < 0.05) compared to the CNT condition (average 16.3 nm) and clustered on a particular size distribution in the contour length as shown Fig 3a. This suggests that structures longer than 20 nm are not favorable, that the aptamer is more compact. This is also supported by the disproportionate decrease of multiple–rip cases. The unfolding–rupture force distributions for the CNT and the Mg^+2^ assays (Fig 3 inset), although not statistically significant (*p* = 0.12), also support the claim.

To gain insight of the APTSTX1 structure, we simulated the aptamer molecule using the conditions described in Materials and Methods. Initially, the aptamer was simulated for 80 ns to obtain its native structure in the absence of ions. Then it went through 50 ns of simulated incubation with different Mg^+2^ ion concentrations (S3 Fig), ranging from 0 − 500 mM. Five replicates for each condition were simulated. As shown in S3a Fig, the radius of gyration is smaller for higher Mg^+2^ concentrations, indicating an increase in folding. Additionally, changes in RMSD and the number of hydrogen bonds within the aptamer further suggest that the aptamer folds more at higher Mg^+2^ concentrations (S3b and S3c Fig) [38]. The number of Mg^+2^ ions within 5 from the aptamer converge to 20 − 40 (S3d Fig).

In CNT conditions, we observed a high variability in the contour length of the APTSTX1 aptamer during rips, suggesting that poorly defined structures were formed. On the other hand, in presence of Mg^+2^ higher-order structures were formed that, although not statistically significant (p = 0.12), appear to shift the unfolding force distribution to higher forces. Furthermore, the contour length of the transitions clustered around a particular value, confirming, from the structural analysis, that Mg^+2^ plays a stabilization role. At higher concentration of Mg^+2^ one main structure is predicted by MFOLD, that was our starting point for MD simulations analysis.

SMD simulations (5 runs of 10 ns each) were done under accelerated conditions to make them time–accessible [37]. The SMD trajectories were analyzed with the same criteria than that of the pulling experiments, giving the rip force and contour length of a constructed 3D Handy aptamer with 1500 ions based on the MFOLD predictions [8]. SMD showed that the rupture force distributions of CNT and Mg^+2^ conditions were relatively similar and that the contour lengths were significantly (*p* < 0.05) smaller for Mg^+2^ conditions (Fig 3b). This observation is in agreement with our data obtained using optical tweezers.

**Fig 4:**
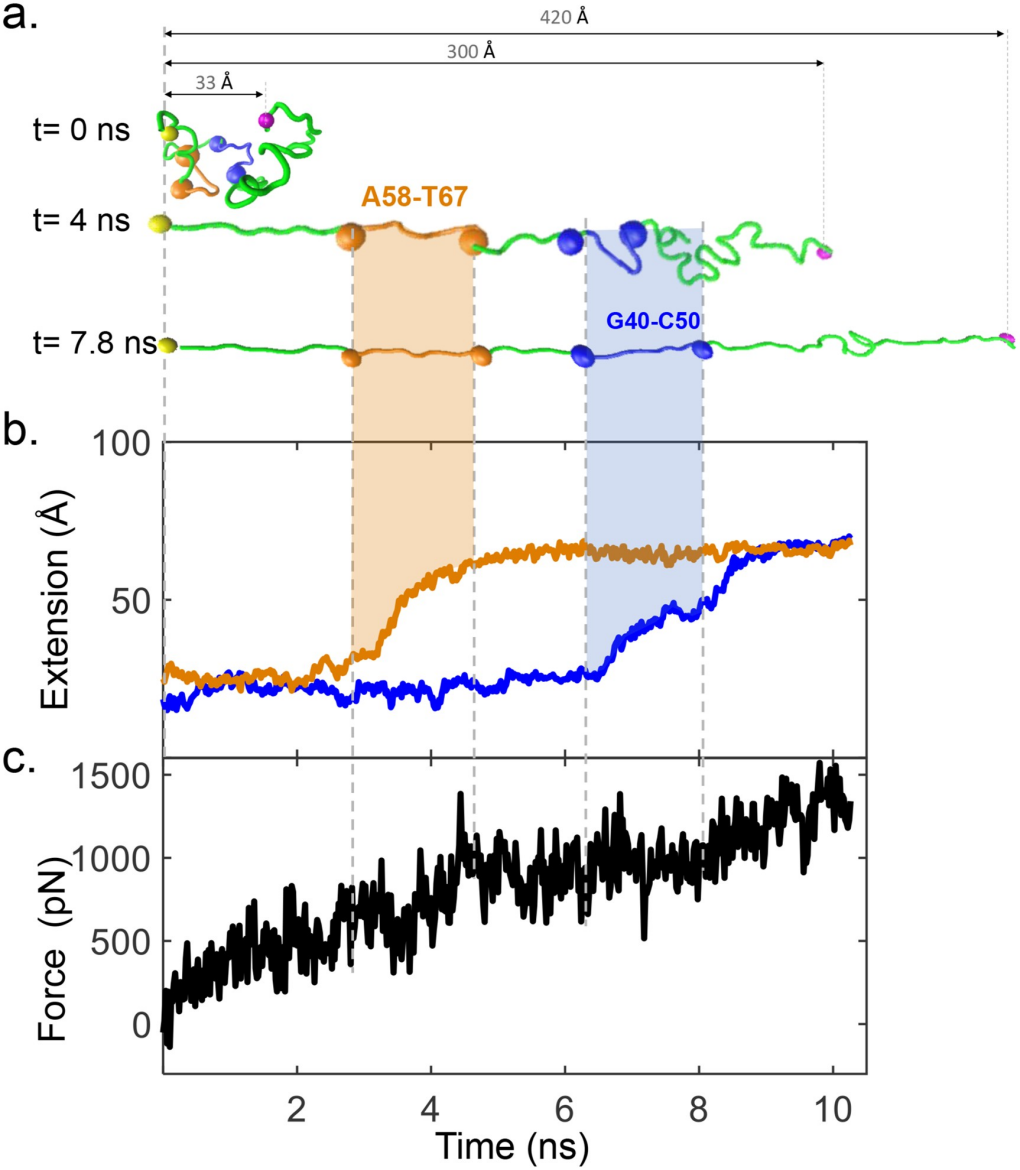
SMD simulations of the APTSTX1 aptamer under Mg^+2^ conditions. (a) Snapshots of SMD simulations highlighting two modelled hairpins in the aptamer corresponding to nucleotides A58–T67 (orange line) and G40–C50 (blue line) paired interactions. Blue and orange spheres represent the nucleotides end pair measured in each hairpin. (b) Distance between nucleotide–paired interactions in the two hairpins modeled via MFOLD (see S4 Fig). Each trajectory represents a representative trace from 5 replicas. SMD simulations show that rips derived from the aptamer depend upon the unfolding of postulated hairpins (A58–T67 and G40–C50) as well as additional novel folded domains.(c) SMD simulation showing the aptamer unfolding force.

Additionally, SMD trajectories showed that rips derived from the aptamer matched the unfolding distance of the postulated hairpins of APTSTX1 [20] (Fig 4) as well as new additional folded domains(Fig 3b). Fig 4a shows a SMD example of two MFOLD-defined hairpins (S4 Fig) formed by nucleotides A58-T67(orange) and G40-C50(blue). Fig 4b shows rips (changes of extension in short time intervals) corresponding to the unfolding first of A58-T67(orange) which is followed by G40-C50(blue). Finally Fig 4c shows the force applied on the molecule.

**Fig 5:**
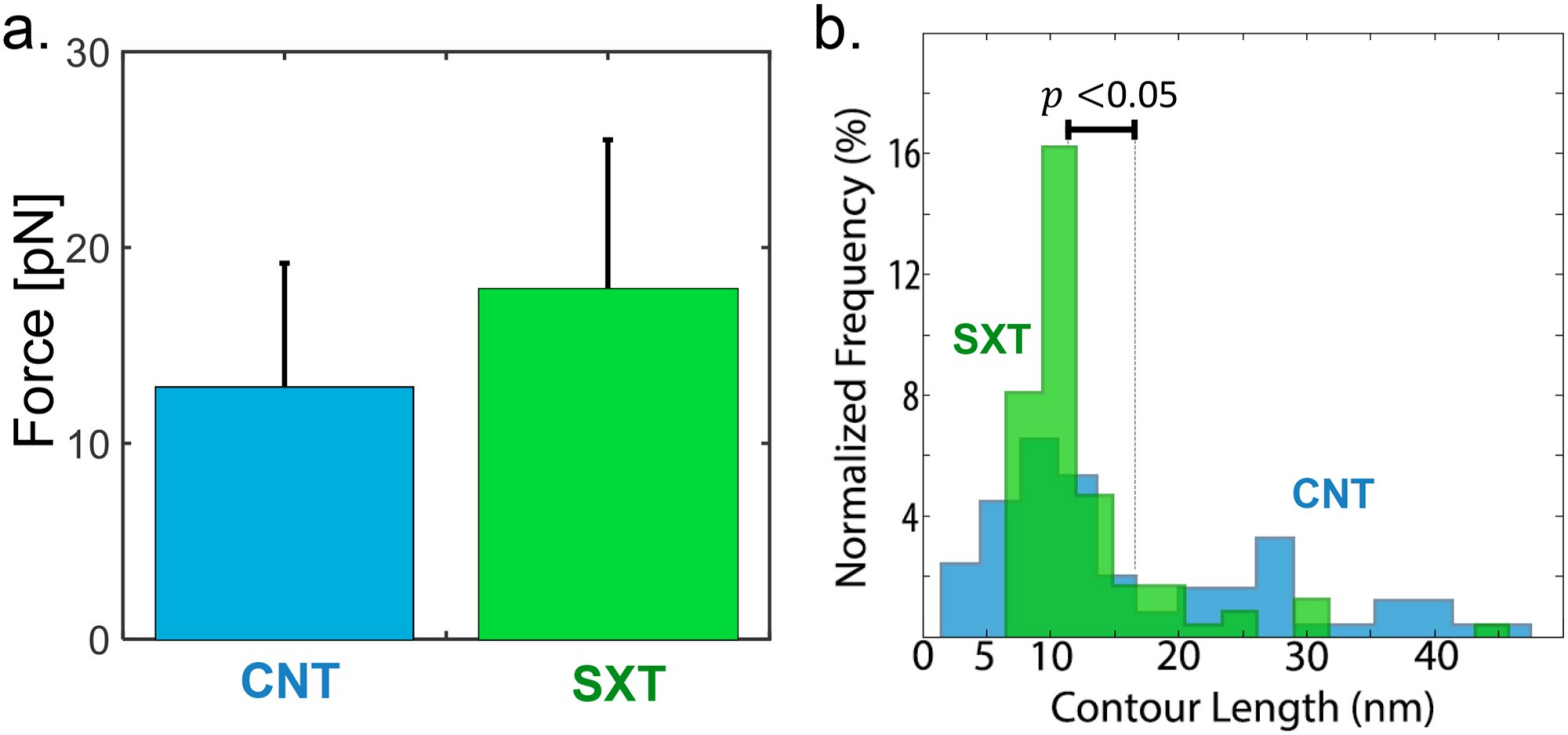
APTSTX1 aptamer stabilization with SXT. (a) Comparison of the rupture force histograms for the control (CNT, blue) and saxitoxin–containing environment (SXT, green). Total number of rips considered (only Unfolding) are CNT: 45, SXT: 40. (b) Comparison of the contour length histograms for the control (CNT, blue) and saxitoxin–containing environment (SXT, green). Total number of rips (Unfolding and Refolding) considered are CNT: 79, SXT: 83.

A global energy minimum was not found within the available computing time for the APTSTX1 aptamer–STX system. Because of this, aptamer pulling experiments in a SXT environment was only performed with optical tweezers. From a total of 77 pulling experiments, we observed smaller structures that clustered in a particular size, in a similar fashion to that observed for Mg^+2^. The contour length distribution (Fig 5) was centered around the average contour length of 12.21 nm. The experiments with STX exhibited single folding transitions in 49.4% of the experiments (Fig 2 SXT), substantially more than the 16.9% for Mg^+2^.

Furthermore, there was a significant increment of the unfolding force in the STX treatment compared to that observed in CNT (*p* < 0.05; Fig 5).

## Conclusion

SMD simulations correlated very well with the clustered contour length at lower extension obtained by optical tweezers. Also the *in silico* and experimental data force distributions were similar between Mg^+2^ and CNT conditions showing that the length distributions represent the most significant change. Additionally, in silico simulations proposed the presence of additional rips different from the initial MFOLD-predicted hairpins that could explain the variability of the numbers of rips in optical tweezer experiments.

The presence of STX affected both force and contour length distributions of the APTSTX1 aptamer, having more prevalent single-rip structures than the CNT case. This suggests the formation of specific structures upon the target presence, in this case, STX. This is expected for these aptamers, particularly those that bind to small molecules [39, 40]. The large broadness of the rip size distribution in CNT compared to that of Mg^+2^, suggests that the aptamer can form many different structures, and that Mg^+2^ stabilizes some of them, as we can conclude from the structural data, albeit, we were not able to get a value of energy stabilization at the single molecule level. More computational power is required to get stable and unbiased bindings of STX molecules, which did not allow us to perform SMD simulations on a SXT–rich environment. However, granted that enough computational power is available, methods developed in this work could be used to simulate the interaction of different aptamers with their targets, including APTSTX1 with SXT.

Once obtaining a definite structure, deduced by SMD and optical tweezer assays, sensors could be engineered based on the APTSTX1 folding structures. There are several advantages of using aptamers on a molecular sensor platform. Aptamers are cost–effective, highly stable and are routinely produced using simple and consistent manufacturing methods, making them promising substitutes for antibodies. The type arrangement of an aptamer on a sensing platform depends on its interaction with the target [18, 41] and the configuration switch from a free–state to a bound–state, given the modification of the tertiary structure when the aptamer binds.

The APTSTX1 aptamer folding configuration estimated by our 3D simulations, and the structural strength and possible location of the hairpins that are broken during the rips allow us to propose strategies to convert this aptamer into a detection device. The change in the force and contour length is significant when using the toxin, after proper standardization, optical tweezers would allow STX detection at the highest sensitivity since the measurement is done from a single molecule perspective. Currently, STX detection is mostly performed by ensemble–averaging approaches, however, successive developments on miniaturization and automation may allow in the future using the single molecule approach as the state of the art of this development. Secondly, further development considering folding strength, could include the addition of a partially complementary ssDNA strand to the reaction, designed as a short sequence, that may disrupt the folded aptamer when in water but not under the folding configuration triggered by its target [42–47]. A double–strand binding fluorophore would exhibit changes in the net fluorescence. Additionally, since the folding of aptamer positions A58 and T67 on the SMD simulations seems to correlate with the findings by using optical tweezers, we estimate that labeling those positions with i.e. pyrene pairs, or with a fluorophore–quencher pair, may allow a wavelength shift upon STX binding, offering another potential biosensor for STX quantification [45, 48, 49]. More studies are needed to assess the effect of other compounds on the binding and on the structural configuration of the APTSTX1 aptamer, and the effect that other ions might exert, considering that a detection device should be stable upon field conditions, and be able to quantify STX on variable mixture compositions in the shellfish extracts used in the mouse bioassay [3, 5–7].

Future detection systems for red tides require bioanalytical approaches that allow higher reproducibility, higher sensitivity and avoid the use of animals. The APTSXT1 aptamer could be used to sensor SXT by exploiting its mechanical changes [50, 51].

## Supporting information

S1 Fig Explanation Diagram of the contour length histogram determination. a) Diagram Force extension curves obtained by pulling the DNA handles with aptamer. (b) Graph of Rip Force vs Rip extension of aptamer. (c) Five Force extension curves obtained by unfolding of aptamer. (d) Five points of Rip Force vs Rip extension obtained of (c). (e)Fits of the worm-like chain(WLC) to representative rip force-extension curves of aptamer to measured rip force of n unfolding events.(f)Histograms of the contour length calculated direct of the WLC model.
(EPS)

S2 Fig Representative curve between trap position and rip force. (a) Control(CNT). (b)Mg^+2^. (c) SXT.
(EPS)

S3 Fig Analysis of the APTSTX1 aptamer via MD simulations after different concentrations of Mg^+2^. (a) Radius of gyration of the aptamer, (b) RMSD of the aptamer, (c) Number of Mg^+2^ ions within 5 of the aptamer and (d) Number of hydrogen bonds intra aptamer. Light-gray, emerald, light-blue and blue lines and symbols represent 0, 15, 150 and 1500 Mg^+2^ ions, respectively.
(EPS)

S4 Fig MD simulations of the Handy aptamer. a) 2D representation of the aptamer structure based on MFOLD, (b) Non-constrained aptamer all-atom model after 80 ns of MD simulations (c) Constrained 3D model to match the 2D aptamer representation (d) MD simulations of the constrained aptamer model after 50 ns.
(EPS)

S1 File Support data 1. Data repository, aptamer control condition and aptamer under SXT.
(ZIP)

S2 File Support data 2. Data repository, aptamer under magnesium.
(ZIP)
